# Blood circulating cell-free mitochondrial DNA as a potential biomarker for major depressive disorder: a meta-analysis

**DOI:** 10.1038/s41398-026-03865-2

**Published:** 2026-02-06

**Authors:** Yaman Zhang, Mingzhe Zhao, Shijie Song, Qiyuan Chen, Yi Meng, Xueli Yu, Wei Wei, Wei Deng, Wanjun Guo, Tao Li, Xueyu Qi

**Affiliations:** 1 Department of Neurobiology, Affiliated Mental Health Center & Hangzhou Seventh People’s Hospital, Zhejiang University School of Medicine, Hangzhou, Zhejiang China; 2 Nanhu Brain-computer Interface Institute, Hangzhou, China; 3 NHC and CAMS Key Laboratory of Medical Neurobiology, MOE Frontier Science Center for Brain Science and Brain-machine Integration, School of Brain Science and Brain Medicine, Zhejiang University, Hangzhou, Zhejiang China; 4 The Fourth School of Clinical Medicine, Zhejiang Chinese Medical University, Hangzhou, Zhejiang 310053 China

**Keywords:** Depression, Diagnostic markers

## Abstract

**Background:**

Mitochondrial dysfunction has been implicated in major depressive disorder (MDD), but reliable, measurable biomarkers remain elusive. As a minimally invasive and quantifiable biomarker, circulating cell-free mitochondrial DNA (ccf-mtDNA) in blood offers potential for objective assessment of mitochondrial stress in MDD. However, evidence linking regarding the association between ccf-mtDNA levels and MDD is limited and inconsistent.

**Methods:**

We systematically searched eight databases, including PubMed, EMBASE, and major Chinese repositories. Thirteen studies with 1370 participants (837 individuals with MDD and 533 controls) were included per PRISMA guidelines. P-values were synthesized using the Lipták-Stouffer Z-score method. Sensitivity and fail-safe N analyses assessed the robustness of the findings and publication bias, and stratified analyses examined the effects of age, antidepressant use, and geographic region.

**Results:**

Across studies, elevated blood ccf-mtDNA levels were significantly associated with MDD (p = 0.013). Stratified analyses revealed stronger associations in older adults (≥60 years old; p = 0.0009), unmedicated patients (p = 4.99 × 10⁻⁶), and North American cohorts (p = 4.29 × 10⁻¹¹), but not in younger individuals (p = 0.83), medicated patients (p = 0.97), and Asian/European samples (p = 0.72, p = 0.99). Sensitivity analyses indicated moderate instability overall but confirmed data robustness in key subgroups.

**Conclusions:**

This is the first meta-analysis to establish a significant link between elevated blood ccf-mtDNA and MDD, highlighting age and antidepressant exposure as critical modulators. These findings support the potential of blood ccf-mtDNA to serve as a biomarker for late-life and drug-naïve depression, with implications for objective diagnosis and personalized treatment.

## Introduction

Major depressive disorder (MDD) is a prevalent and debilitating psychiatric condition characterized by persistent low mood, anhedonia, cognitive impairment, and diminished engagement in daily activities [1]. Globally, MDD is a leading cause of disability, affecting approximately 3.8% of the population and contributing substantially to the overall burden of disease [2]. The onset of the COVID-19 pandemic alarmingly exacerbated the incidence and severity of MDD, underscoring the critical and urgent need for enhanced mental health strategies [3]. Epidemiological studies have revealed the following concerning statistics: approximately 53% of individuals diagnosed with MDD report suicidal ideation, and 31% have attempted suicide [4], highlighting the need for early detection, precise diagnosis, and effective intervention to mitigate this significant risk.

Despite the profound clinical impact of MDD, its diagnosis relies predominantly on subjective clinical interviews and the use of symptom rating scales, such as the Hamilton Depression Rating Scale (HAM-D) [5]. This reliance on subjective assessments represents a fundamental limitation in current psychiatric practice: the absence of objective, quantifiable biomarkers to support diagnosis, prognostic prediction, and treatment monitoring. The identification of reliable biomarkers is a critical unmet need in psychiatric research, with the potential to facilitate earlier detection, improve diagnostic accuracy, and enable the development of personalized treatment approaches.

The pathophysiology of MDD is intricate and multifactorial, and involves a dynamic interplay among genetic, environmental, and neurobiological factors [6]. While traditional hypotheses have focused on monoaminergic dysregulation and hyperactivity of the hypothalamic-pituitary-adrenal (HPA) axis [7, 8], a growing body of evidence now implicates mitochondrial dysfunction as a key contributor to MDD pathology [9]. In this context, “mitochondrial dysfunction” broadly encompasses mitochondrial stress, oxidative damage, or impaired quality-control mechanisms that disturb bioenergetic homeostasis and promote the release of mitochondrial components, such as mitochondrial DNA (mtDNA), into the circulation [10, 11], rather than referring solely to reductions in enzymatic or respiratory capacity. Although the brain constitutes only approximately 2% of the total body weight, it consumes approximately 20% of the body’s oxygen and glucose to sustain synaptic activity, neurotransmission, and neuroplasticity [12]. Owing to limited intrinsic energy reserves, the brain is critically dependent on mitochondrial oxidative phosphorylation to meet its high ATP demand [13]. Consequently, impairments in mitochondrial bioenergetics, particularly reduced ATP synthesis, have been increasingly associated with various psychiatric disorders, including MDD [14].

A critical consequence of mitochondrial dysfunction is the overproduction of reactive oxygen species and reactive nitrogen species, leading to a state of oxidative stress [15–17]. mtDNA is particularly vulnerable to this stress because of its lack of protective histones, high replication rate, and limited repair capacity [18, 19]. Damaged mtDNA can be released into the cytoplasm and subsequently into the bloodstream via extracellular vesicles or other cellular pathways, forming circulating cell-free mitochondrial DNA (ccf-mtDNA) [20].

Importantly, ccf-mtDNA, as an indirect indicator of mitochondrial stress or impaired quality control, functions as a damage-associated molecular pattern, capable of activating pattern recognition receptors such as Toll-like receptor 9 (TLR9) on microglia [21, 22]. This activation promotes the release of proinflammatory cytokines and neurotoxic mediators, thereby establishing a link between mitochondrial dysfunction and neuroinflammation [23], which is increasingly recognized as a process central to the pathogenesis of MDD. As a circulating and quantifiable byproduct of mitochondrial damage, ccf-mtDNA is promising as a readily accessible peripheral biomarker that reflects mitochondrial health and, by extension, has potential utility in MDD diagnosis and treatment monitoring [24]. Despite the promising role of ccf-mtDNA as a biomarker, current research findings remain inconsistent. A previous meta-analysis reported no significant difference in blood ccf-mtDNA levels between individuals with MDD and healthy controls [25], thus raising questions about the robustness and reproducibility of the association between ccf-mtDNA levels and MDD.

To resolve the inconsistencies in previous findings, we conducted a comprehensive meta-analysis to examine the association between blood ccf-mtDNA levels and MDD. We adopted a broad inclusion strategy to capture diverse sources of evidence, synthesizing findings using the Lipták-Stouffer Z-score method, an established approach that allows the integration of p-values across studies with heterogeneous effect sizes and sample characteristics [26–30]. In addition, we performed subgroup analyses based on antidepressant usage, age, and geographic region to identify potential sources of heterogeneity. These factors were selected due to their biologically and environmentally relevance to mitochondrial regulation. For instance, antidepressant exposure may alter mitochondrial bioenergetics [31], aging is associated with accumulated mitochondrial damage and impaired quality control [32], and mitochondrial DNA haplogroups and heteroplasmy patterns vary across populations, influencing mitochondrial function and mtDNA release dynamics [33]. Moreover, regional environmental and lifestyle factors (e.g., diet, pollution, stress exposure) may further contribute to the variability observed across studies. By integrating methodological rigor with a comprehensive analytical framework, the findings of this study offer novel insights into the role of mitochondrial dysfunction in depression and critically evaluates the potential of ccf-mtDNA to be used as a peripheral biomarker to support the achievement of an objective diagnosis and personalized treatment for people diagnosed with MDD.

## Materials and methods

### Study registration

This meta-analysis was performed in accordance with the Preferred Reporting Items for Systematic Reviews and Meta-Analyses (PRISMA) guidelines [34]. The review protocol was registered with PROSPERO under the registration number CRD420250653914.

### Literature search

**Fig. 1:**
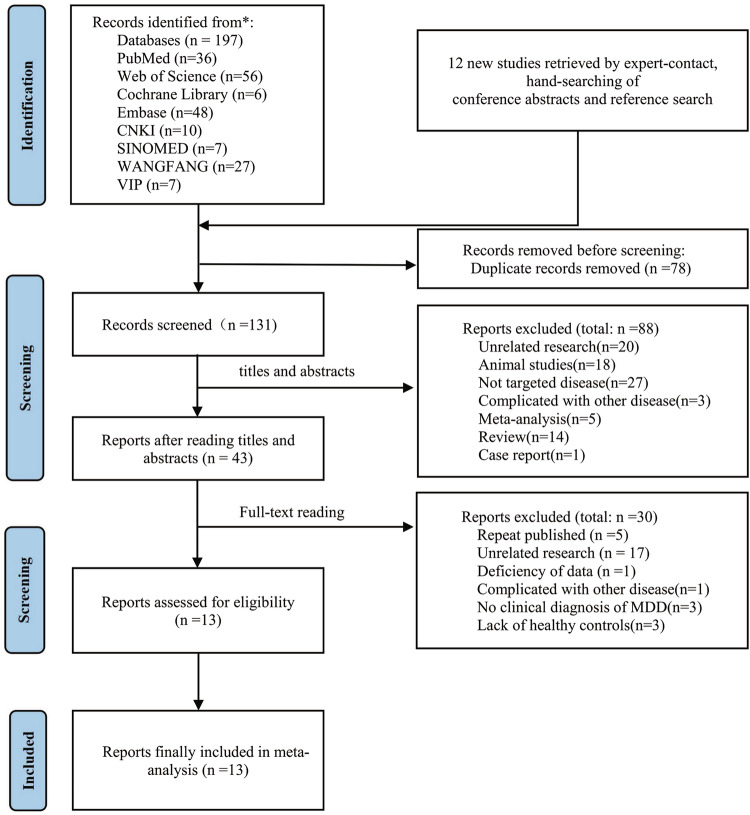
Flow diagram of study screening process.

We conducted a comprehensive search across four English-language electronic databases (PubMed, Web of Science, Cochrane Library, and EMBASE) and four Chinese literature databases (CNKI, SINOMED, Wanfang, and the VIP Database). The search used the following keywords: (“ccf-mtDNA” OR “cell-free mitochondrial DNA” OR “circulating mitochondrial DNA”) AND (“Depressive Disorder” OR “depression”). The search spanned from 1975–April 2025. To ensure comprehensive coverage, we also reviewed the reference lists of relevant reviews and meta-analyses, identifying 12 studies pertinent to our topic. This process was independently conducted by two researchers. Ultimately, 13 independent studies that met the inclusion criteria were included, with 1370 participants (837 individuals with MDD and 533 healthy controls). The study selection process is depicted in Fig. 1.

To explore the impacts of the study design characteristics, we conducted stratified analyses using three approaches. First, the studies were grouped by the mean age of participants as follows: the young and middle-aged group (16–59 years) and the older group (≥60 years). Second, the studies were stratified based on whether participants had received an antidepressant medication within three months prior to the assessment and blood sampling. Third, subgroup analyses were performed by geographic region, with the United States and Canada categorized as North America, Germany and Sweden categorized as Europe, and Japan and China categorized as Asia.

### Inclusion and exclusion criteria

The inclusion criteria were as follows: (1) studies included patients with MDD and healthy controls; (2) studies measured ccf-mtDNA levels in blood and reported p values for differences between the groups; and (3) when duplicate datasets were identified during full-text screening, the study with the most comprehensive results was prioritized. The exclusion criteria were as follows: (1) duplicate studies; (2) studies lacking a healthy control group or including controls that were not confirmed to be free of MDD according to clinical diagnostic criteria.

### Quality assessment

Two authors independently evaluated the quality of the included studies using the Newcastle-Ottawa Scale (NOS; developed by Wells et al., Ottawa Hospital Research Institute, available at http://www.ohri.ca/programs/clinical_epidemiology/oxford.asp), which assesses study quality across three domains: selection, comparability, and exposure/outcome. Consistent with the established principles and prior research [26–28], we did not weight studies based on their quality score or exclude low-scoring studies. The quality assessment results are available in Supplementary Table 1 for reader reference.

### P-value extraction

P-values were independently extracted by two authors from each study without discrepancies. For studies reporting imprecise p-values (e.g., p < 0.01), we contacted the corresponding authors to obtain exact values. If the attempt was unsuccessful, the maximum reported p-value (e.g., p = 0.01) was used. When multiple p-values were reported in the same study because of the use of varying statistical methods, we selected the p value adjusted for confounding factors.

### Statistical analysis

We used the Lipták-Stouffer Z-score method to combine significance levels across heterogeneous studies, as many reports presented diverse test statistics and analytical approaches that precluded direct pooling of standardized effect sizes. This method integrates *P*-values with sample-size weighting to provide an overall test of association, but it does not estimate pooled effect sizes and therefore precludes calculation of conventional heterogeneity indices such as I² or Cochran’s Q. Accordingly, we conducted sensitivity analyses (leave-one-out) and visually inspected the distribution of study-specific Z-scores to evaluate the consistency of findings across studies.

The direction of each study’s effect was explicitly incorporated during the conversion of two-tailed to one-tailed *P*-values, based on whether the reported results were consistent with the hypothesis that elevated ccf-mtDNA levels are associated with MDD. Specifically, *P* < 0.50 indicated higher ccf-mtDNA levels in the MDD group, whereas *P* > 0.50 indicated higher levels in the control group. The one-tailed *P*-values were then transformed into Z-scores, assigned positive values for *P* < 0.05 and negative values for *P* > 0.05. The weighted combined Z-score (Z_w_) was calculated using the following formula [26–30, 35]:

$${Z}_{w}=\frac{{\sum }_{i=1}^{k}{W}_{i}{Z}_{i}}{\sqrt{{\sum }_{i=1}^{k}{W}_{i}^{2}}}$$

where Zi represents the z-score of each study, Wi denotes the sample size as the weighting factor, and k is the total number of studies. The resulting (Z_w_) follows a standard normal distribution, with corresponding probabilities derived from a normal distribution table. This approach was applied to the overall analysis and analysis of stratified subgroups.

To assess robustness of the results, sensitivity analyses were conducted by systematically excluding one study at a time and recalculating Z_w_, evaluating the influence of individual studies on the overall findings. Publication bias was examined by calculating the fail-safe N for both the overall and stratified analyses. Following previous studies [26–30], we estimated the number of additional studies with a p-value of 0.50 and the average sample size required to render the weighted Lipták-Stouffer result nonsignificant. Additionally, the ratio of the fail-safe N to the number of included studies was computed to gauge the potential impact of publication bias.

## Results

### Overall analysis

Our literature search identified 209 potentially relevant studies. After screening the titles and abstracts, 43 studies were selected for full-text review. Ultimately, 13 studies, including three conference abstracts, met all the inclusion criteria and were included in the meta-analysis. The study selection process is detailed in Fig. 1, and the characteristics of the included studies are presented in Table 1.

**Table 1:** Characteristics of all studies included in the Meta-Analysis.

Study	location	NO. of participants	Age, mean (years)	Use antidepressant (Yes/No)	Sample	Averaged 1-tailed P value	Lipták-Stouffer P value after study exclusion
Kageyama et al. [47]	Japan	129	44.9	No	Plasma	0.9999995	7.54 × 10^−5^
Lindqvist et al. [51]	USA	184	NS	No	Plasma	0.000005	0.31
Lindqvist et al. [31]	USA	105	38.6	No	Plasma	0.0005	0.06
Ampo et al. [52]	Canada	53	69.3	No	Plasma	0.005	0.03
Fernström et al. [46]	Sweden	285	37.9	Yes	Plasma	0.9995	1.48 × 10^−8^
Gonçalves et al. [53]	Canada	53	69.0	No	Plasma	0.0025	0.03
Ampo et al. [54]	Canada	39	68.9	No	Plasma	0.007	0.02
Behnke et al. [55]	Germany	44	30.8	Yes	Serum	0.1905	0.01
Mendes-Silva et al. [56]	Canada	90	NS	NS	Plasma	0.0235	0.03
Zhou et al. [57]	Canada	33	16.4	No	Plasma	0.6525	0.01
Daniels et al. [58]	Mix	109	46.6	Yes	Plasma	0.0015	0.06
Mendes-Silva et al. [59]	Canada	90	69.4	No	Plasma	0.485	0.01
Jin et al. [60]	China	156	24.0	No	Plasma	0.0005	0.12
**Total:**	**1370**						
**Average sample size:**	**105**					**0.013**	

**Fig. 2:**
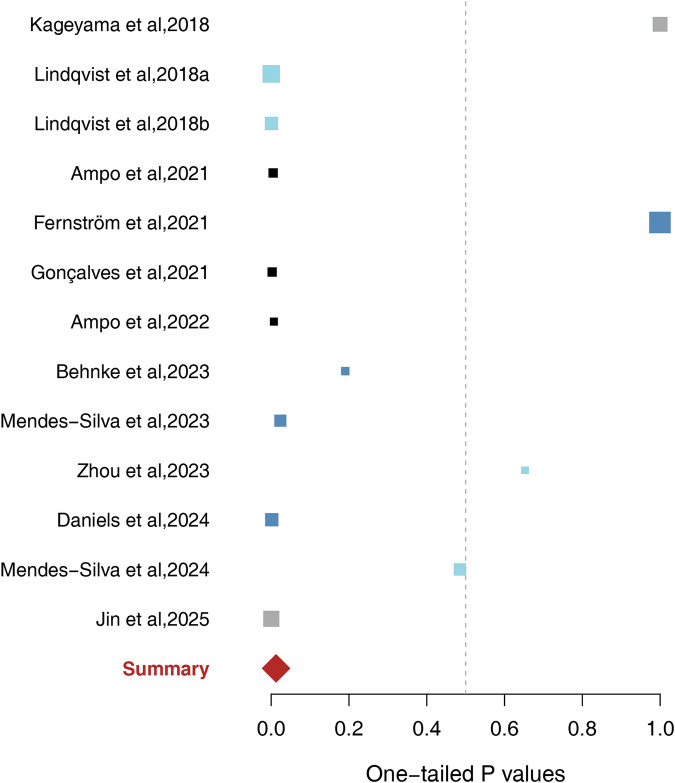
Forest plot of 13 human studies regarding blood ccf-mtDNA in depression. The squares mark represents the one-tailed P value for each study: p < 0.50 indicates higher ccf-mtDNA levels in depression, whereas p > 0.50 suggests higher ccf-mtDNA levels in controls. The size of the box reflects relative sample size. The red diamond denotes the overall result of meta-analysis. Dark gray squares mark studies that all included subjects had not been treated with antidepressants; sky-blue indicates studies conducted in North America that included antidepressant-naïve participants; and black indicates studies conducted in North America that included participants aged over 60 without using antidepressants. All studies presented in the forest plots correspond to those listed in Table 1, where full reference citations are provided.

These 13 studies, with 1370 participants (837 individuals with MDD and 533 healthy controls), were pooled to assess the association between blood ccf-mtDNA levels and MDD. The combined analysis revealed a significant association between elevated ccf-mtDNA levels and MDD (p = 0.013) (Fig. 2). Sensitivity analysis showed that the overall p-value ranged from 1.48 × 10⁻⁸ to 0.3 when each study was sequentially excluded (Table 1). To render the overall result nonsignificant, more than 83 unpublished or undiscovered studies with an average sample size of 105 and a non-significant result (p = 0.50) would be needed, yielding a fail-safe ratio of approximately 6 unreported studies per included study. Notably, when the analysis was restricted to the five studies that were included in a previous meta-analysis on this topic, no significant associations were observed (p = 0.96) (Supplementary Table 2), which is consistent with prior findings.

### Subgroup analysis by age

**Fig. 3:**
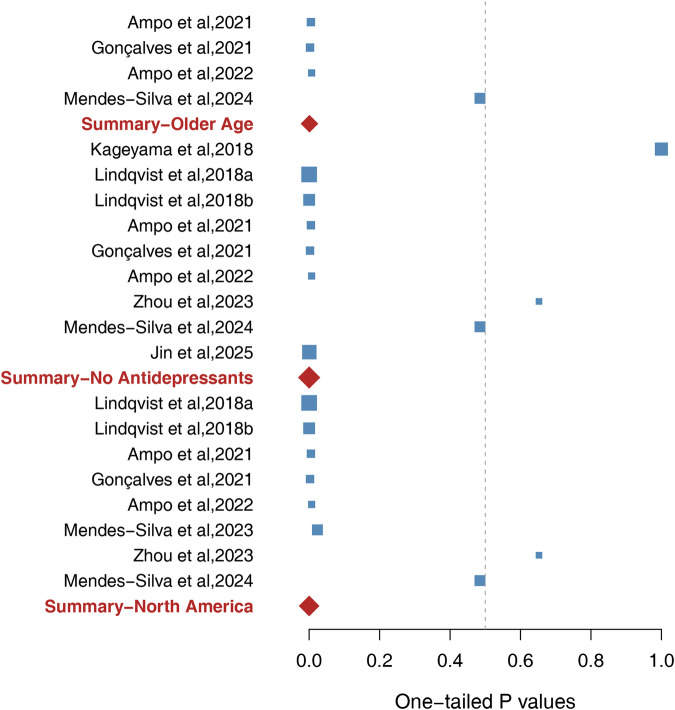
Forest plot of 21 human studies regarding blood ccf-mtDNA in subgroups of Late-Life Depression, drug naïve MDD and depression in North America. The squares mark represents the one-tailed P value for each study: p < 0.50 indicates higher ccf-mtDNA levels in depression, whereas p > 0.50 suggests higher ccf-mtDNA levels in controls. The size of the box reflects relative sample size. The three red diamonds represent the overall meta-analytic results for the older age group, participants without antidepressant treatment in the past three months, and North American group, respectively.

Four studies, involving 235 participants, focused exclusively on older adults (≥60 years) or analyzed older cohorts separately. A significant association was found between elevated blood ccf-mtDNA levels and late-life MDD (p = 0.0009) (Fig. 3). Sensitivity analysis confirmed the robustness of this result, with p-values ranging from 3.23 × 10⁻⁶ to 0.007 upon the sequential removal of each study (Table 2). To render this result non-significant, more than 19 unpublished studies with null effects (p = 0.50) and an average sample size of 59 would be needed, corresponding to a fail-safe ratio of approximately 5 unreported studies per included study. In contrast, seven studies involving 861 young and middle-aged participants (16–59 years) revealed no significant associations between elevated blood ccf-mtDNA levels and MDD (p = 0.83) (Supplementary Table 3).

**Table 2:** Studies included in the meta-analysis stratified by age, antidepressant use and geographic region.

Study	NO. of participants	location	Age, mean (years)	Use antidepressant (Yes/No)	Averaged 1-tailed P value	Lipták-Stouffer P value after study exclusion
***Older age group***						
Ampo et al. [52]	53	Canada	69.3	No	0.005	0.01
Gonçalves et al. [53]	53	Canada	69.0	No	0.0025	0.02
Ampo et al. [54]	39	Canada	68.9	No	0.007	0.007
Mendes-Silva et al. [59]	90	Canada	69.4	No	0.485	3.23 × 10^−6^
**Total:**	**235**					
**Average sample size:**	**59**				**0.0009**	
***Non-antidepressants group***						
Kageyama et al. [47]	129	Japan	44.9	No	0.9999995	1.42 × 10^−12^
Lindqvist et al. [51]	184	USA	NS	No	0.000005	0.01
Lindqvist et al. [31]	105	USA	38.6	No	0.00001	2.06 × 10^−4^
Ampo et al. [52]	53	Canada	69.3	No	0.005	2.60 × 10^−5^
Gonçalves et al. [53]	53	Canada	69.0	No	0.0025	3.07 × 10^−5^
Ampo et al. [54]	39	Canada	68.9	No	0.007	1.67 × 10^−5^
Zhou et al. [57]	33	Canada	16.4	No	0.6525	3.70 × 10^−6^
Mendes-Silva et al. [59]	90	Canada	69.4	No	0.485	2.19 × 10^−6^
Jin et al. [60]	156	China	24.0	No	0.0005	6.38 × 10^−4^
**Total:**	**842**					
**Average sample size:**	**94**				**4.99** **×** **10**^**−6**^	
***North American group***						
Lindqvist et al. [51]	184	USA	NS	No	0.000005	9.79 × 10^−7^
Lindqvist et al. [31]	105	USA	38.6	No	0.00001	8.20 × 10^−9^
Ampo et al. [52]	53	Canada	69.3	No	0.005	5.43 × 10^−10^
Gonçalves et al. [53]	53	Canada	69.0	No	0.0025	7.30 × 10^−10^
Ampo et al. [54]	39	Canada	68.9	No	0.007	2.93 × 10^−10^
Mendes-Silva et al. [56]	90	Canada	NS	NS	0.0235	3.13 × 10^−10^
Zhou et al. [57]	33	Canada	16.4	No	0.6525	2.18 × 10^−11^
Mendes-Silva et al. [59]	90	Canada	69.4	No	0.485	2.74 × 10^−12^
**Total:**	**647**					
**Average sample size:**	**81**				**4.29** **×** **10**^**−11**^	

### Subgroup analysis by antidepressant use

Nine studies, with 842 participants, included patients who had not used antidepressants within the past three months or had never used them. The pooled analysis revealed a significant association between elevated blood ccf-mtDNA levels and MDD (p = 4.99 × 10⁻⁶) (Fig. 3). This association remained significant upon the removal of any single study (1.42 × 10⁻¹² ≤ p ≤ 0.01) (Table 2). To overturn this result, 57 unpublished studies with an average sample size of 93 and nonsignificant findings (p = 0.50) would be needed, yielding a fail-safe ratio of approximately 6 unreported studies per included study. Conversely, three studies involving 438 participants, in which patients were either using antidepressants or whose antidepressant use was not specified, showed no significant association (p = 0.97) (Supplementary Table 4).

### Subgroup analysis by geographic region

Eight studies, involving 647 participants from North America (United States and Canada), demonstrated a strong association between elevated blood ccf-mtDNA levels and MDD (p = 4.29 × 10⁻¹¹) (Fig. 3). This result remained significant even after any single study was excluded (2.74 × 10⁻¹² ≤ p ≤ 9.79 × 10⁻⁷) (Table 2). To render this finding nonsignificant, more than 105 unpublished studies with null effects (p = 0.50) and an average sample size of 81 would be needed, which corresponded to a fail-safe ratio of approximately 13 unreported studies per included study. In contrast, stratified analyses for Asia (p = 0.72) and Europe (p = 0.99) revealed no significant associations (Supplementary Table 5).

## Discussion

This meta-analysis, which synthesized data from 13 independent studies, provided the most robust evidence to date for a significant association between elevated blood ccf-mtDNA levels and MDD. Our findings support the hypothesis that mitochondrial dysfunction plays a central role in MDD pathophysiology. While blood ccf-mtDNA does not represent mitochondrial activity within a single cell type, it serves as a systemic marker of mitochondrial stress, likely originating from metabolically active cells such as leukocytes, endothelial cells, and platelets, which are particularly sensitive to oxidative and inflammatory stress [36]. Subgroup analyses revealed that this association was particularly pronounced among older adults (≥60 years old), antidepressant-naïve patients, and individuals from North America, suggesting that ccf-mtDNA levels may be modulated by age, pharmacological status, and geographic or population-specific factors. Collectively, these results highlight the potential for ccf-mtDNA to serve as a biomarker and to be used to achieve stratified diagnoses and precision psychiatric treatment for people diagnosed with MDD.

Mitochondrial dysfunction has been increasingly recognized as a core pathological feature of MDD [37–39]. As a peripheral, minimally invasive, and quantifiable indicator of mitochondrial health, blood ccf-mtDNA is a promising tool for use in clinical settings. In contrast to a previous meta-analysis, which was based on five studies and reported no significant associations [25], our expanded analysis, including a larger sample size (1370 participants) and stratified analyses to reduce heterogeneity, yielded statistically significant results. Nonetheless, sensitivity analyses revealed some instability in that the removal of certain individual studies attenuated the significance of the overall findings. This underscores the need for further investigation into factors that may confound or modulate ccf-mtDNA levels.

### Age-related effects

The significant association between elevated ccf-mtDNA levels and MDD, which was observed in older adults, but not in younger or middle-aged individuals, suggests a potential age-specific vulnerability. This may reflect a synergistic interaction between aging-related mitochondrial decline and depression-induced oxidative stress. Aging is known to impair oxidative phosphorylation [40] and the autophagic clearance of damaged mitochondria [41], whereas MDD contributes to chronic oxidative stress and the inflammatory burden [15–17]; together these effects of aging and MDD may promote the release of mtDNA into the circulation [41]. In contrast, younger individuals may retain more efficient mitochondrial quality control mechanisms [32], including the stress-induced upregulation of mitophagy-related proteins, such as Beclin-1 and ATG5 [42], thereby limiting ccf-mtDNA accumulation. However, because most included studies did not report detailed clinical variables such as depression severity, age at onset, or duration of illness, we were unable to evaluate their potential influence on ccf-mtDNA levels. Future studies that integrate standardized measures of symptom severity and illness chronicity will be crucial for elucidating these effects and refining the interpretation of age-related differences. These findings suggest that blood ccf-mtDNA levels may serve as an age-stratified biomarker and highlight the need for longitudinal studies across the lifespan, especially in adolescents, to elucidate the developmental and temporal dynamics of ccf-mtDNA levels in relation to MDD onset.

### Influence of antidepressant use

Our subgroup analysis revealed a significant association between elevated blood ccf-mtDNA levels and MDD in unmedicated patients, but not in those with recent antidepressant exposure. These findings are consistent with evidence that antidepressants can modulate mitochondrial function [43]. For example, selective serotonin reuptake inhibitors (SSRIs) at therapeutic doses may enhance mitochondrial bioenergetics by upregulating mitofusin-2 (MFN2) and promoting mitochondrial repair pathways [44].However, at supratherapeutic concentrations, some SSRIs may inhibit mitochondrial respiratory complexes I and IV, leading to further mitochondrial damage [45]. The absence of a significant association between ccf-mtDNA levels and MDD in medicated patients may therefore reflect the protective or masking effects of antidepressants on mitochondrial stress. For example, Fernström et al., the largest included study, reported lower ccf-mtDNA levels in individuals with depression who had received antidepressant treatment prior to blood collection [46]. This decrease may indicate a normalization of mitochondrial stress induced by effective pharmacotherapy rather than an unreported confounding effect. In contrast, Kageyama et al., which examined unmedicated patients, also observed reduced ccf-mtDNA levels [47], suggesting that assay differences, sample size, or illness-stage effects (e.g., early versus chronic MDD) might underlie the opposite direction of association rather than medication status per se. In addition, circulating mtDNA may exist in both vesicle-bound and free forms [48], reflecting distinct biological pathways of release, active secretion through extracellular vesicles or passive leakage resulting from cell injury or apoptosis, which could further contribute to inter-study variability in measured ccf-mtDNA concentrations. Notably, our results for the unmedicated subgroup remained robust in sensitivity analyses, indicating that ccf-mtDNA may be a specific marker for antidepressant-naive MDD patients. Future studies should rigorously document the medication status and incorporate longitudinal sampling to assess the predictive utility of ccf-mtDNA levels for the treatment response and disease progression.

### Geographic variation

We also detected substantial geographic variation with a strong association between blood ccf-mtDNA levels and MDD was observed in studies conducted in North America, but not in those from Asia or Europe. While these findings may partially reflect limited statistical power due to there being fewer studies from outside North America, underlying biological and sociocultural differences may also contribute. For example, racial and ethnic differences in baseline ccf-mtDNA levels have been reported; in one study, Black heart transplant recipients had significantly higher ccf-mtDNA levels in plasma than white patients did, which was correlated with worse clinical outcomes [49]. Additionally, mitochondrial haplogroups linked to ancestral origins can affect mtDNA replication, the damage response, and release dynamics [33]. In addition to genetic and ancestral factors, variations in lifestyle and metabolic health may also contribute to the geographic disparities observed in ccf-mtDNA levels. For instance, higher body mass index (BMI) and Western dietary patterns, characterized by high caloric intake and processed foods, have been linked to oxidative stress and chronic inflammation, both of which can increase mitochondrial damage and the release of mtDNA into the circulation [50]. Such population-level differences in metabolic and inflammatory burden may therefore partly explain the elevated ccf-mtDNA levels observed in North American cohorts. Together, these findings underscore the need to interpret ccf-mtDNA levels within population-specific reference frameworks and to validate their biomarker potential across cohorts with diverse ethnicities and dietary patterns.

### Strengths and limitations

This study has several strengths. It represents the most comprehensive synthesis to date, incorporating 13 studies which is more than twice the number included in the dataset used in the previous meta-analysis [25]. Nevertheless, several limitations should be acknowledged. First, the reliance on P values from primary studies may have introduced bias, particularly from smaller samples with limited statistical power. Second, because the Lipták-Stouffer Z-score method integrates significance levels rather than standardized effect sizes, conventional heterogeneity indices (e.g., I², Q) could not be estimated. Third, differences in quantification methods, biospecimen type (plasma vs. serum), and pre-analytical handling may contribute to variability in mtDNA measurements, underscoring the need for standardized collection and processing protocols in future studies. Moreover, the lack of methodological harmonization across cohorts and the potential influence of physiological factors (e.g., circadian rhythm, hormonal fluctuations, systemic inflammation) may further limit measurement accuracy and cross-study comparability, thereby constraining the translational applicability of ccf-mtDNA as a biomarker. Finally, the cross-sectional design of most included studies prevented causal inference; whether elevated ccf-mtDNA represents a cause, consequence, or correlate of MDD remains uncertain. These limitations highlight important directions for future research.

## Conclusion

This meta-analysis of 13 studies involving 1370 participants provided compelling evidence for a significant association between elevated blood ccf-mtDNA levels and MDD, reinforcing the central role of mitochondrial dysfunction in MDD. Stratified analyses identified age and antidepressant use as key modulators of this association, explaining previous inconsistencies and positioning ccf-mtDNA as a promising candidate biomarker, particularly in older adults and antidepressant-naïve individuals. These findings pave the way for the development of objective diagnostic tools and personalized treatment strategies for MDD. To fully establish the diagnostic specificity, prognostic value, and clinical utility of ccf-mtDNA in monitoring treatment responses, future research should prioritize large-scale, multicenter studies with standardized measurement protocols, ethnically diverse populations, and longitudinal designs.

## Supplementary information

Supplementary Table 1；Supplementary Table 2；Supplementary Table 3；Supplementary Table 4；Supplementary Table 5

## Data Availability

All data in this meta-analysis was publicly available.
