# Supplementary material for: Blood circulating cell-free mitochondrial DNA as a potential biomarker for major depressive disorder: a meta-analysis
Source: Transl Psychiatry. 2026 Feb 6;16:83. doi: 10.1038/s41398-026-03865-2 (PMC12923772; doi:10.1038/s41398-026-03865-2)
Supplement: Supplementary file 1 — Supplementary Table 1；Supplementary Table 2；Supplementary Table 3；Supplementary Table 4；Supplementary Table 5 [file 41398_2026_3865_MOESM1_ESM.docx]

**Supplementary Table 1.** Quality assessment of studies.

| Score  Study | Selection | | | | Comparability | Exposure | | | Total  score |
| --- | --- | --- | --- | --- | --- | --- | --- | --- | --- |
|  | Definition of the cases | Representativeness of the cases | Selection of Controls | Definition of Controls | Comparability of cases and controls on the basis of the design or analysis | Ascertainment of exposure | Same method of ascertainment for cases and controls | Non-Response rate |  |
| Kageyama et al.  (2018) | 1 | 1 | 0 | 1 | 1 | 1 | 1 | 1 | 7 |
| Lindqvist et al.  (2018b) | 1 | 1 | 1 | 1 | 2 | 1 | 1 | 0 | 8 |
| Fernström et al.  （2021） | 1 | 1 | 1 | 1 | 1 | 1 | 1 | 1 | 8 |
| Gonçalves et al.  （2021） | 1 | 1 | 0 | 1 | 2 | 1 | 1 | 0 | 7 |
| Ampo et al.  (2022) | 1 | 1 | 0 | 1 | 1 | 1 | 1 | 0 | 6 |
| Behnke et al.  (2023) | 1 | 1 | 1 | 0 | 1 | 1 | 1 | 1 | 7 |
| Mendes-Silva et al. (2023) | 1 | 0 | 0 | 0 | 2 | 1 | 1 | 1 | 6 |
| Zhou et al.  (2023) | 1 | 1 | 0 | 1 | 1 | 1 | 1 | 0 | 6 |
| Daniels et al.  (2024) | 0 | 1 | 1 | 1 | 2 | 1 | 1 | 0 | 7 |
| Mendes-Silva et al. (2024) | 1 | 1 | 0 | 1 | 2 | 1 | 1 | 0 | 7 |
| Jin et al.  (2025) | 1 | 1 | 1 | 1 | 1 | 1 | 1 | 1 | 8 |

**Supplementary Table 2.** Studies included in the previous meta-analysis.

| **Study** | **NO. of participants** | **Age, mean**  **(years)** | **Averaged 1-tailed P value** | **Lipták-Stouffer P**  **value after study**  **exclusion** |
| --- | --- | --- | --- | --- |
| Lindqvist et al. (2016) | 74 | 38.5 | 1.49 × 10^-12^ | 0.999592558 |
| Lindqvist et al. (2018b) | 105 | 38.6 | 0.00001 | 0.999367464 |
| Kageyama et al. (2018) | 138 | 44.9 | 0.9999995 | 0.418459737 |
| Fernström et al. (2021) | 285 | 37.9 | 0.9995 | 0.045242576 |
| Behnke et al. (2023) | 44 | 30.8 | 0.1905 | 0.971158571 |
| **Total:** | **646** |  |  |  |
| **Average sample size:** | **129** |  | **0.96** |  |

**Supplementary Table 3.** Studies included in the young and middle-aged group meta-analysis.

| **Study** | **NO. of participants** | **Age, mean**  **(years)** | **Averaged 1-tailed P value** | **Lipták-Stouffer P**  **value after study**  **exclusion** |
| --- | --- | --- | --- | --- |
| Kageyama et al. (2018) | 129 | 44.9 | 0.9999995 | 0.20 |
| Lindqvist et al. (2018b) | 105 | 38.6 | 0.0005 | 0.97 |
| Fernström et al. (2021) | 285 | 37.9 | 0.9995 | 0.01 |
| Behnke et al. (2023) | 44 | 30.8 | 0.1905 | 0.85 |
| Zhou et al. (2023) | 33 | 16.4 | 0.6525 | 0.80 |
| Daniels et al. (2024) | 109 | 46.6 | 0.0015 | 0.97 |
| Jin et al. (2025) | 156 | 24.0 | 0.0005 | 0.99 |
| **Total:** | **861** |  |  |  |
| **Average sample size:** | **123** |  | **0.83** |  |

**Supplementary Table 4.** Studies included in antidepressants group meta-analysis.

| **Study** | **NO. of participants** | **Use antidepressant**  **(Yes/No)** | **Averaged 1-tailed P value** | **Lipták-Stouffer P**  **value after study**  **exclusion** |
| --- | --- | --- | --- | --- |
| Fernström et al. (2021) | 285 | Yes | 0.9995 | 0.001 |
| Behnke et al. (2023) | 44 | Yes | 0.1905 | 0.978 |
| Daniels et al. (2024) | 109 | Yes | 0.0015 | 0.999 |
| **Total:** | **438** |  |  |  |
| **Average sample size:** | **146** |  | **0.97** |  |

**Supplementary Table 5.** Studies included in Asian and European group meta-analysis.

| **Study** | **location** | **NO. of participants** | **Averaged 1-tailed P value** | **Lipták-Stouffer P**  **value after study**  **exclusion** |
| --- | --- | --- | --- | --- |
| ***Asian group*** |  |  |  |  |
| Kageyama et al. (2018) | Asia | 129 | 0.9999995 | 0.0005 |
| Jin et al. (2025) | Asia | 156 | 0.0005 | 0.9999995 |
| **Total:** | **285** |  |  |  |
| **Average sample size:** | **143** |  | **0.72** |  |
|  |  |  |  |  |
| ***European group*** |  |  |  |  |
| Fernström et al. (2021) | Europe | 285 | 0.9995 | 0.1905 |
| Behnke et al. (2023) | Europe | 44 | 0.1905 | 0.9995 |
| **Total:** | **329** |  |  |  |
| **Average sample size:** | **165** |  | **0.99** |  |
